# Multimorbidity, frailty and polypharmacy in European and Asian patients with atrial fibrillation: a comparison of two regional prospective observational registries

**DOI:** 10.1007/s11357-025-02026-5

**Published:** 2025-12-04

**Authors:** Davide Antonio Mei, Marco Proietti, Giulio Francesco Romiti, Tommaso Bucci, Bernadette Corica, Alena Shantsila, Hung-Fat Tse, Giuseppe Boriani, Tze-Fan Chao, Gregory Y. H. Lip

**Affiliations:** 1 Cardiology Division, Department of Biomedical, Metabolic and Neural Sciences, University of Modena and Reggio Emilia, Policlinico Di Modena, Modena, Italy; 2 Clinical and Experimental Medicine PhD Program, University of Modena and Reggio Emilia, Modena, Italy; 3 Liverpool Centre for Cardiovascular Science at University of Liverpool, Liverpool John Moores University and Liverpool Heart & Chest Hospital, Liverpool, UK; 4 Department of Clinical Sciences and Community Health, University of Milan, Milan, Italy; 5 Division of Cardiogeriatric Subacute Care, IRCCS Istituti Clinici Scientifici Maugeri, Milan, Italy; 6 Department of Translational and Precision Medicine, Sapienza – University of Rome, Rome, Italy; 7 Department of General Surgery and Surgical Specialties “Paride Stefanini”, Sapienza University of Rome, Rome, Italy; 8 Division of Cardiology, Department of Medicine, School of Clinical Medicine, Queen Mary Hospital, The University of Hong Kong, Hong Kong, Hong Kong SAR China; 9 Division of Cardiology, Department of Medicine, Taipei Veterans General Hospital, Taipei, Taiwan; 10 Institute of Clinical Medicine, and Cardiovascular Research Center, National Yang Ming Chiao Tung University, Taipei, Taiwan; 11 Danish Center for Health Services Research, Department of Clinical Medicine, Aalborg University, Aalborg, Denmark; 12 Medical University of Bialystok, Bialystok, Poland

**Keywords:** Atrial fibrillation, Clinical complexity, Europe, Asia

## Abstract

Multimorbidity, frailty, and polypharmacy are associated with worse outcomes in patients with atrial fibrillation (AF), leading to ‘clinically complex’ patient phenotypes. Possible differences between European and Asian patients regarding these aspects have not been studied. We studied AF patients derived from two large prospective observational AF registries, conducted in Europe and Asia. Multimorbidity and polypharmacy were defined according to the number of comorbidities and drugs at baseline. Frailty was defined according to a 40-items frailty index (FI). Prescription of OAC was assessed at baseline. The primary outcome was the composite of all-cause death and major adverse cardiovascular events. European patients had a higher burden of multimorbidity, frailty, and polypharmacy domains compared with Asians. Asian patients with these domains were less likely to be prescribed OAC than Europeans, especially those who were frail. After adjustments, being frail was associated with lower OAC prescription, with Asians less likely prescribed than Europeans (OR 0.34, 95% CI 0.25–0.45 vs. OR 0.47, 95% CI 0.40–0.55, p_int_ = 0.037). Adjusted Cox regression found that multimorbidity, frailty, and polypharmacy domains were associated with a higher risk of the composite outcome. On subgroup analysis, frail Asian patients had a higher risk of the composite outcome (p_int_ = 0.007) than Europeans. Multimorbidity, frailty and polypharmacy have different epidemiological characteristics amongst European and Asian AF patients. Being frail was associated with a higher likelihood of not being prescribed OAC, particularly in Asian patients. The adverse impact of ‘clinically complex’ patient phenotypes on risks of adverse outcomes was greater in Asian patients than in Europeans.

**Graphical Abstract:**

**Figure:**
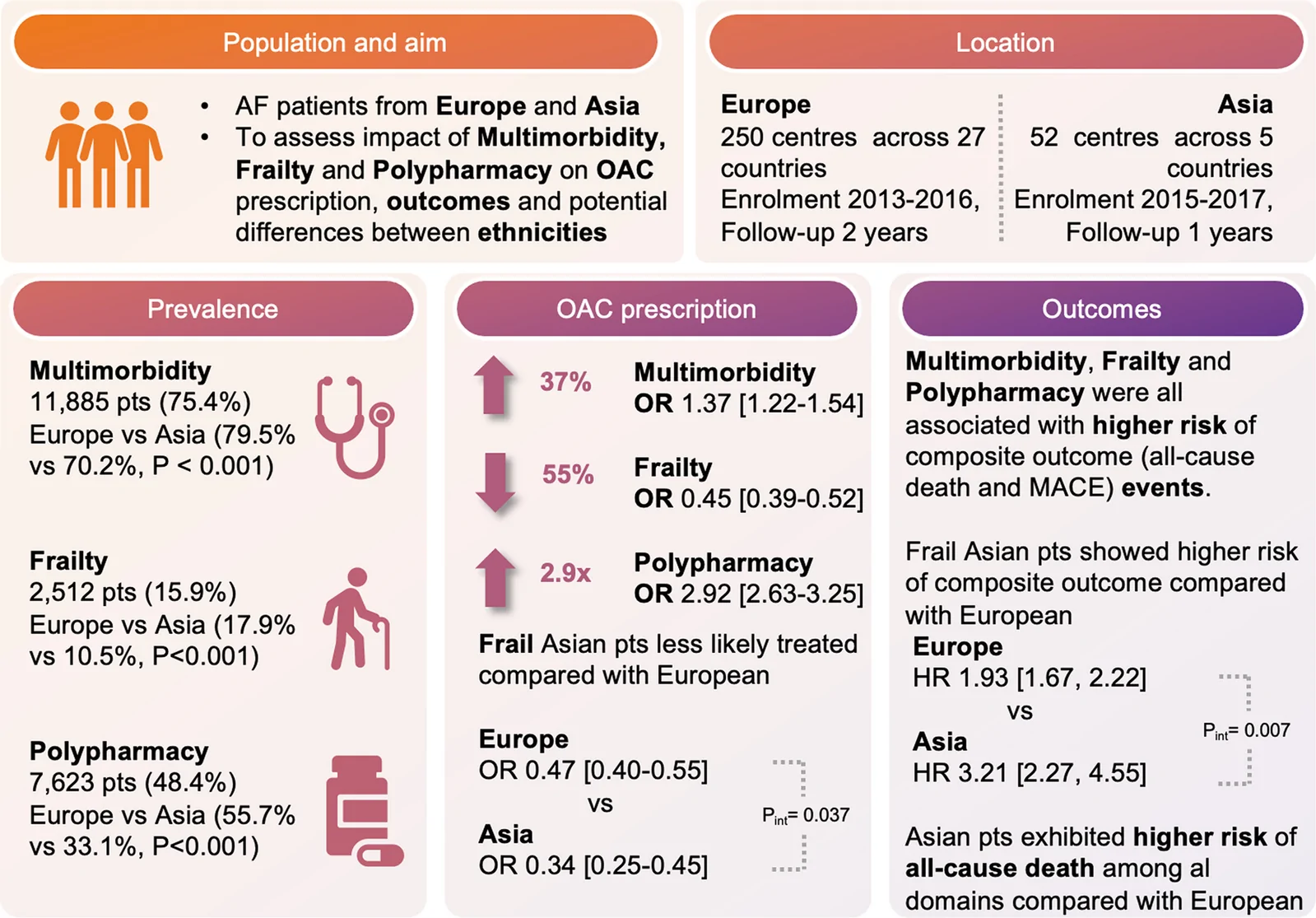

**Supplementary Information:**

The online version contains supplementary material available at https://doi.org/10.1007/s11357-025-02026-5.

## Introduction

The global prevalence of atrial fibrillation (AF) is increasing, in parallel with an increasingly elderly population and a progressive increase in the burden of chronic conditions affecting these individuals [1–3]. More effective risk stratification and broader adoption of oral anticoagulation have led to a reduction in stroke-related mortality, which now represents fewer than 10% of deaths in patients with AF [4]. Nevertheless, a residual risk of stroke and other cardiovascular events remains despite anticoagulation [5, 6], and the steady increase in non-cardiovascular hospitalization highlights the growing relative impact of associated comorbidities in these patients [7].

Considering these factors, the contemporary AF management has shifted toward a more holistic and integrated approach that encompasses a wide range of cardiovascular and non-cardiovascular comorbidities that have been shown to substantially influence clinical trajectories and therapeutic decisions in increasingly elderly AF individuals [8, 9]. These comorbidities frequently coexist and interact, contributing to different ‘clinically complex’ phenotypes with implications for management and outcomes in AF patients [10–12]. Indeed, in recent years, the concept of ‘clinically complex’ phenotypes has gained growing attention in AF research, specifically addressing three overlapping yet distinct constructs and domains, i.e. multimorbidity, frailty, and polypharmacy [13–15]. These three entities are commonly referred to as key domains of clinical complexity, each encompassing specific dimensions (e.g. disease burden, functional status, medication load) and being influenced by multiple determinants such as age, socioeconomic status, and healthcare access.

Nevertheless, the impact of multimorbidity, frailty, and polypharmacy on treatment patterns and outcomes remains only partially addressed, particularly in non-Western countries. The aims of this study conducted in two large prospective European and Asian AF cohorts were to: i) assess the prevalence and epidemiological distribution of multimorbidity, frailty, and polypharmacy among European and Asian individuals; ii) investigate their association with antithrombotic treatment and major clinical outcomes; and iii) compare these associations between European and Asian populations.

## Methods

### Study population

We analyzed data from two large prospective observational cohorts of patients with AF; one conducted in Europe and the other in Asia. The methodology, baseline characteristics, and main findings of these registries have been previously described in detail [16]. In brief, both studies enrolled consecutive adult patients (aged > 18 years) with electrocardiographically confirmed AF diagnosed within the 12 months preceding enrolment. Written informed consent was obtained from all participants.

The European cohort was derived from the EURObservational Research Programme (EORP) Atrial Fibrillation General Long-Term Registry, which included patients from 250 centers across 27 countries between October 2013 and September 2016, with a follow-up of 2 years [17]. Ethical approval was obtained at both national and institutional levels, and the study complied with the Declaration of Helsinki and EU guidelines for Good Clinical Practice (CPMP/ECH/135/95).

The Asian cohort was derived from the Asia–Pacific Heart Rhythm Society (APHRS) AF registry, which enrolled patients from 52 sites across five countries, primarily in Southeast Asia. Recruitment took place between late 2015 and early 2017, and participants were followed for one year [18]. The respective local ethics committees approved the study protocol.

### Study procedures

Baseline demographic, clinical, and treatment data were collected through a standardized electronic case report form (eCRF). Importantly, both registries used an identical electronic case report form to collect patients’ data. For clarity and simplicity throughout the manuscript, we refer to individuals enrolled in the European registry as “European patients” and those from the Asian registry as “Asian patients.” This terminology reflects the source registry and not necessarily the ethnic or geographic identity of each participant.

Thromboembolic and bleeding risks were evaluated using the CHA₂DS₂-VASc/CHA₂DS₂-VA [19, 20] and HAS-BLED [21] scores, respectively. The investigator reported the presence of the different comorbidities based on direct clinical evaluation and/or medical records, when available. The use of oral anticoagulants (OACs), other antithrombotic therapies, as well as other treatments, was recorded at baseline after enrolment.

### Definitions of multimorbidity, frailty, and polypharmacy

Multimorbidity was defined as the coexistence of two or more chronic comorbidities among the list of 12 examined at baseline, in line with the current definition in the literature [22]. Frailty was assessed using a 40-item frailty index (FI) as reported in a previous analysis [23] and constructed according to the cumulative deficit model originally proposed by Rockwood and Mitnitski [24]. The FI was based on a multidimensional assessment including patients’ vital signs, comorbidities, symptoms, biomarkers, and functional measures. Details regarding the items used are reported in Supplementary Table S1. For each participant, the FI was calculated as the ratio between the number of deficits present and the total number of deficits considered (*n* = 40). Patients were included in the analysis if data were available for at least 85% of the 40 items; the FI denominator was adjusted according to the number of available variables for each patient, in accordance with the methodology reported in the literature [25]. The presence of frailty was defined as a frailty index ≥ 0.25. Polypharmacy was defined as the concomitant use of five or more medications at baseline, in line with the majority of previous studies in the literature [26].

### Follow-up and major adverse events

According to the original design of the two registries, patients from the European cohort were followed for a planned period of 2 years, while those in the Asian cohort were followed for 1 year. In this analysis, the primary endpoint was a composite of all-cause death and major adverse cardiovascular events (MACEs). MACEs were defined as the occurrence of any thromboembolic event (ischemic stroke or systemic embolism), any acute coronary syndrome, or cardiovascular death. Secondary exploratory endpoints included the individual components of the primary endpoint—namely, all-cause death and MACEs—as well as major bleeding events.

As an additional exploratory analysis for the outcome assessment, we also evaluated the cumulative burden of complexity (specifically, the presence of one, two, or all three domains of multimorbidity, frailty, and polypharmacy) as compared to patients without any of these domains.

### Statistical analysis

Continuous variables were reported as median and interquartile range (IQR) and compared across groups using the Kruskal–Wallis test. Categorical variables were presented as numbers and percentages and compared using the Chi-square test.

We used multivariable logistic regression models to assess the association between each domain of clinical complexity (i.e., multimorbidity, frailty, and polypharmacy) and the prescription of OAC at baseline. Time-to-event analyses for the primary and secondary outcomes were performed using Kaplan–Meier curves, with survival distributions compared via the log-rank test. To account for the different follow-up durations between the European and Asian cohorts, incidence rates (IR per 100 person-years with 95% confidence intervals) were calculated for all outcomes. Multivariable Cox proportional hazards models were used to evaluate the association between clinical complexity domains and adverse outcomes. As a secondary exploratory analysis, we also examined the cumulative burden of clinical complexity by evaluating the number of domains present (i.e., 1, 2, or 3 vs. none, of multimorbidity, frailty, and polypharmacy) in relation to outcomes. Interaction analyses were conducted in both logistic and Cox models to assess potential effect modification by region of enrolment (Europe vs. Asia).

Both logistic and Cox regression models were adjusted for age, sex, CHA₂DS₂-VASc scores (HAS-BLED score when studying the association with major bleeding), type of AF, and EHRA class; Cox models were additionally adjusted for baseline OAC therapy. The same covariates were applied in the interaction analyses. Results were reported as Odds Ratios (ORs) or Hazard Ratios (HRs) with 95% Confidence Intervals (CIs). A two-sided *p*-value < 0.05 was considered statistically significant. All analyses were conducted using R software version 4.0.3 (R Foundation for Statistical Computing, Vienna, Austria).

## Results

### Baseline characteristics

**Fig. 1:**
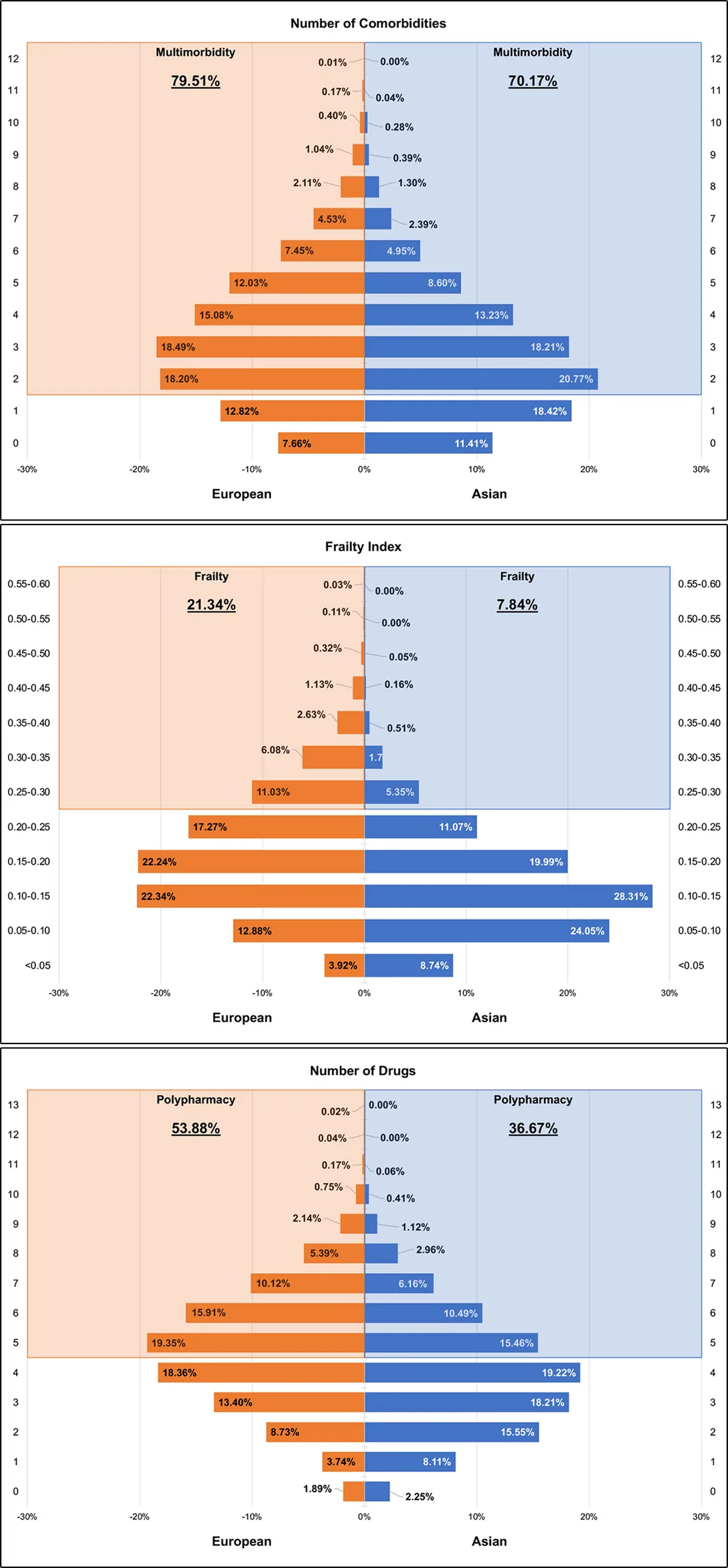
Distribution of Comorbidities, Frailty Index, and Number of Drugs Between European and Asian Patients

Among the 15,762 patients included in the study, 11,885 (75.4%) were classified as multimorbid, with a higher prevalence among European patients compared to Asian patients (79.5% vs 70.2%, *P* < 0.001). A total of 2,512 patients were classified as frail, with a higher prevalence in European patients compared to Asian ones (17.9% vs 10.5%, *P* < 0.001). Polypharmacy was reported in 7,623 patients, again more common in European than in Asian patients (55.7% vs 33.1%, *P* < 0.001). The distribution of the overall cohort according to the number of comorbidities, drugs, and FI is shown in Fig. 1.

Table 1 summarizes the differences between European and Asian patients according to the presence of each domain of multimorbidity, frailty, and polypharmacy. European patients more commonly reported permanent AF, were more significantly symptomatic, and showed a greater burden of thromboembolic and bleeding risk, as reflected by higher baseline CHA₂DS₂-VASc/CHA₂DS₂-VA and HAS-BLED scores, irrespective of the clinical complexity domain.

**Table 1:** Baseline Characteristics of the population according to Multimorbidity, Frailty and Polypharmacy and stratified by the registries

	Multimorbidity		Frailty		Polypharmacy	
	European *N* = 8655	Asian *N* = 3230	European *N* = 2172	Asian *N* = 340	European *N* = 5927	Asian *N* = 1696
Age, (years) (median [IQR])	72.00 [64.50, 78.00] _*0.108*_	71.00 [64.00, 79.00]_*0.108*_	73.00 [66.00, 79.00]	76.00 [68.00, 84.00]	72.00 [65.00, 78.00] _*0.677*_	71.00 [64.00, 79.00] _*0.677*_
Female, *n* (%)	3641 (42.1)	1159 (35.9)	1074 (49.4) _*0.142*_	153 (45.0) _*0.142*_	2551 (43.0)	649 (38.3)
BMI (median [IQR])°	27.70 [24.80, 31.20]	24.80 [22.40, 27.70]	28.90 [25.50, 32.70]	25.10 [22.30, 28.00]	28.00 [25.20, 31.60]	25.40 [22.80, 28.25]
AF type, *n* (%) #						
First diagnosed	1205 (14.1)	230 (7.1)	325 (15.1)	33 (9.7)	803 (13.7)	121 (7.2)
Paroxysmal	2054 (24.1)	1214 (37.6)	504 (23.4)	97 (28.5)	1364 (23.3)	585 (34.6)
Persistent	1587 (18.6)	741 (23.0)	397 (18.5)	65 (19.1)	1079 (18.4)	434 (25.7)
Long-standing persistent	392 (4.6)	329 (10.2)	114 (5.3)	54 (15.9)	268 (4.6)	145 (8.6)
Permanent	3286 (38.5)	711 (22.0)	811 (37.7)	91 (26.8)	2335 (39.9)	407 (24.1)
Cardiovascular disease						
Hypertension, *n* (%)	6221 (72.0)	2447 (75.9)	1742 (80.6)	303 (89.6)	4242 (72.1)	1333 (78.9)
Diabetes mellitus, *n* (%)	2420 (28.1)	1065 (33.2)	949 (43.8)	190 (56.2)	2044 (34.7)	694 (41.2)
Dyslipidemia, *n* (%) #	4134 (49.5)_*0.894*_	1590 (49.7)_*0.894*_	1247 (59.2)	243 (71.9)	2923 (51.4)	1002 (59.4)
CAD, *n* (%) #	2915 (35.9)	837 (26.4)	1025 (51.3) _*0.191*_	186 (55.4) _*0.191*_	2270 (41.3)	559 (33.4)
Heart Failure, *n* (%)	4156 (48.3)	940 (29.5)	1586 (73.6)	203 (61.1)	3068 (52.2)	579 (34.8)
Previous TE events, *n* (%)	1139 (13.2) _**0.005**_	487 (15.2) _**0.005**_	409 (19.0) _**0.001**_	90 (26.8) _**0.001**_	787 (13.4)	253 (15.0) _*0.092*_
PAD, *n* (%) #	853 (10.1)	58 (1.8)	386 (18.4)	17 (5.1)	626 (10.8)	34 (2.0)
Comorbidities						
CKD, *n* (%)	1328 (15.5)	344 (10.7)	664 (30.7) _**0.035**_	123 (36.6) _**0.035**_	1012 (17.2)	208 (12.3)
Anemia, *n* (%)	581 (6.7)	324 (10.0)	336 (15.5)	130 (38.3)	407 (6.9)	198 (11.7)
Malignancy (current + prior), *n* (%)	761 (8.9) _**0.003**_	344 (10.7) _**0.003**_	242 (11.2)	79 (23.2)	441 (7.5) _**0.036**_	154 (9.1) _**0.036**_
Previous bleeding events, *n* (%)	500 (5.8)	311 (9.7)	219 (10.2)	90 (26.8)	360 (6.1)	169 (10.0)
Educational status, *n* (%) $						
Primary school (or less)	1968 (30.7)	896 (39.0)	558 (33.0)	136 (53.3)	1385 (31.3)	538 (41.9)
Domestic Status, *n* (%) §						
Living alone	1288 (17.7)	283 (10.5)	359 (19.1)	35 (11.0)	148 (10.0)	
CHA_2_DS_2_-VASc (median [IQR])	4.00 [2.00, 5.00]	3.00 [2.00, 4.00]	4.00 [3.00, 5.00] _**0.002**_	5.00 [4.00, 6.00] _**0.002**_	4.00 [3.00, 5.00]	3.00 [2.00, 5.00]
CHA_2_DS_2_-VA (median [IQR])	3.00 [2.00, 4.00]	3.00 [2.00, 4.00]	4.00 [3.00, 5.00]	4.00 [3.00, 5.00]	3.00 [2.00, 4.00]	3.00 [2.00, 4.00]
HAS-BLED (median [IQR])	2.00 [1.00, 2.00]	1.00 [1.00, 2.00]	2.00 [1.00, 3.00]	2.00 [2.00, 3.00]	2.00 [1.00, 2.00] _**0.001**_	2.00 [1.00, 2.00] _**0.001**_
EHRA score 3–4, *n* (%)	1755 (20.3)	185 (5.7)	902 (41.5)	70 (20.6)	1256 (21.2)	101 (6.0)
Any OAC, *n* (%)	7535 (87.1)	2733 (84.6)	1801 (83.0) _**0.003**_	259 (76.2) _**0.003**_	5449 (91.9) _**0.001**_	1516 (89.4) _**0.001**_
Any rate control, *n* (%)	6813 (78.9)	2183 (67.8)	1791 (82.7) _**0.001**_	254 (74.9) _**0.001**_	5207 (87.9)	1426 (84.1)
IC & III AADs, *n* %	2229 (25.8)	649 (20.2)	607 (28.1)	52 (15.3)	1816 (30.6)	365 (21.5)

Legend: *all comparisons depict statistically significant differences at *p* < 0.001, except those that were explicitly reported in the subscripts, in which Italic characters depict non-significant differences and Bold characters depict significant differences, All variables presented had less than 1% missing data, unless otherwise specified as follows: # 1–5%; ° 5–10%; § 10–20%; $ > 20%, *AADs*, antiarrhythmic drugs; *AF*, atrial fibrillation; *BMI*, body mass index; *CAD*, coronary artery disease; *EHRA*, European Heart Rhythm Association; *IQR*, interquartile range; *PAD*, peripheral artery disease; *OAC*, oral anticoagulation; *TE*, thromboembolic events

Regarding the presence of baseline comorbidities, European patients with any domain of multimorbidity, frailty, or polypharmacy had a higher prevalence of coronary artery disease, heart failure, peripheral artery disease, and chronic kidney disease. In comparison, Asian ones had a higher prevalence of hypertension, diabetes mellitus, dyslipidemia (except for multimorbidity), anemia, malignancy, and prior thromboembolic or bleeding events, particularly in the frail subgroup.

Asian patients were more likely to have attained only primary education or less, whereas they were less likely to live alone compared to European patients.

### Multimorbidity, frailty, and polypharmacy domains and OAC prescription

Differences in the prescription of pharmacological therapies according to clinical complexity domains and across the registries are reported in Supplementary Table S2. In the overall cohort, multimorbidity and polypharmacy were associated with higher odds of OAC prescription at baseline, while frailty was associated with lower odds of prescription (Table 2).

**Table 2:** Relationship between Multimorbidity, Frailty and Polypharmacy Domains,, OAC use and Differences across the Registries

	OR [95% CI]	*P*-value	P for interaction
Multimorbidity			
Overall	1.37 [1.22–1.54]	< 0.001	
*Subgroup*			
European	1.34 [1.17–1.54]	< 0.001	0.698
Asian	1.40 [1.18–1.65]	< 0.001	
Frailty			
Overall	0.45 [0.39–0.52]	< 0.001	
*Subgroup*			
European	0.47[0.40–0.55]	< 0.001	**0.037**
Asian	0.34 [0.25–0.45]	< 0.001	
Polypharmacy			
Overall	2.92 [2.63–3.25]	< 0.001	
*Subgroup*			
European	3.19 [2.81–3.61]	< 0.001	**0.004**
Asian	2.31 [1.92–2.78]	< 0.001	

*OAC*, oral anticoagulation: Legend *CI*, Confidence interval; *OR*, Odds Ratio

Concerning differences between European and Asian patients, no significant interaction was observed for multimorbidity (Table 2). Frailty was associated with lower OAC use among Asian patients compared to European patients (p_int_ = 0.037). Polypharmacy was associated with higher odds of being prescribed OAC in Europe than in Asia (p_int_ = 0.004).

### Follow-up and adverse outcomes

**Fig. 2:**
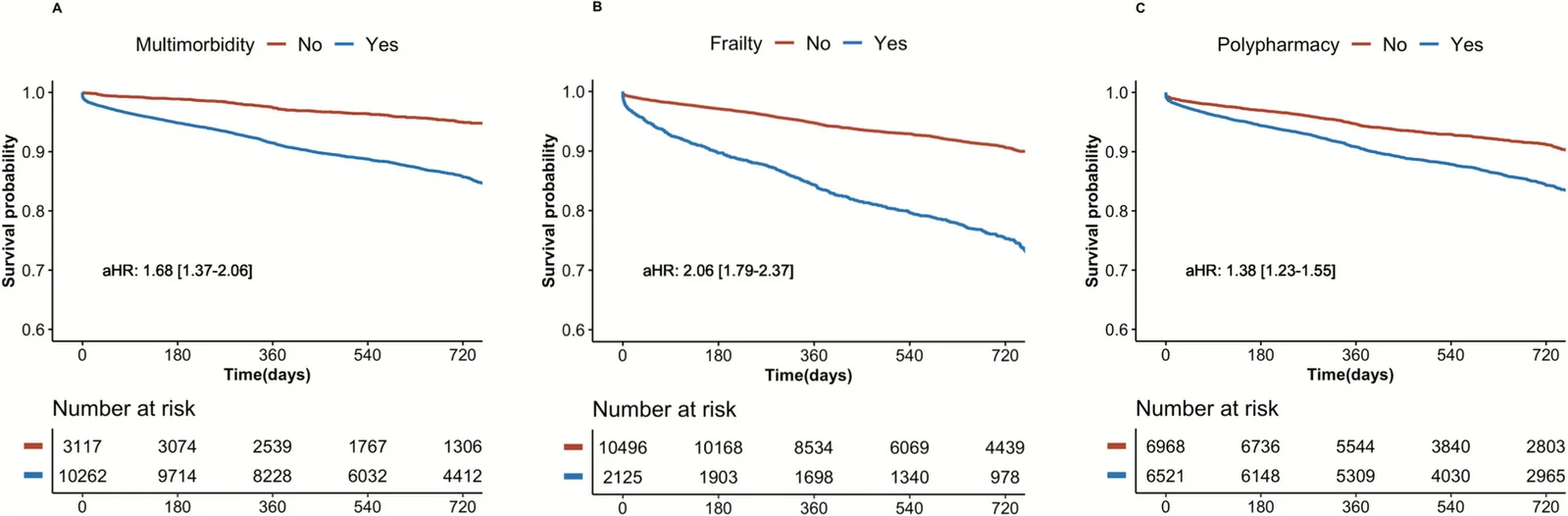
Kaplan–Meier curves plotting the survival probability for the Multimorbidity, Frailty and Polypharmacy domains, alongside the adjusted hazard ratio obtained by the Cox regression model

After a median follow-up of 694 days [IQR 365–735], Kaplan–Meier curves showed that patients presenting with clinical complexity domains had a significantly higher cumulative incidence of the primary composite outcome in the overall cohort of patients. This association remained statistically significant after adjustment in multivariable Cox proportional hazards models [Fig. 2].

**Fig. 3:**
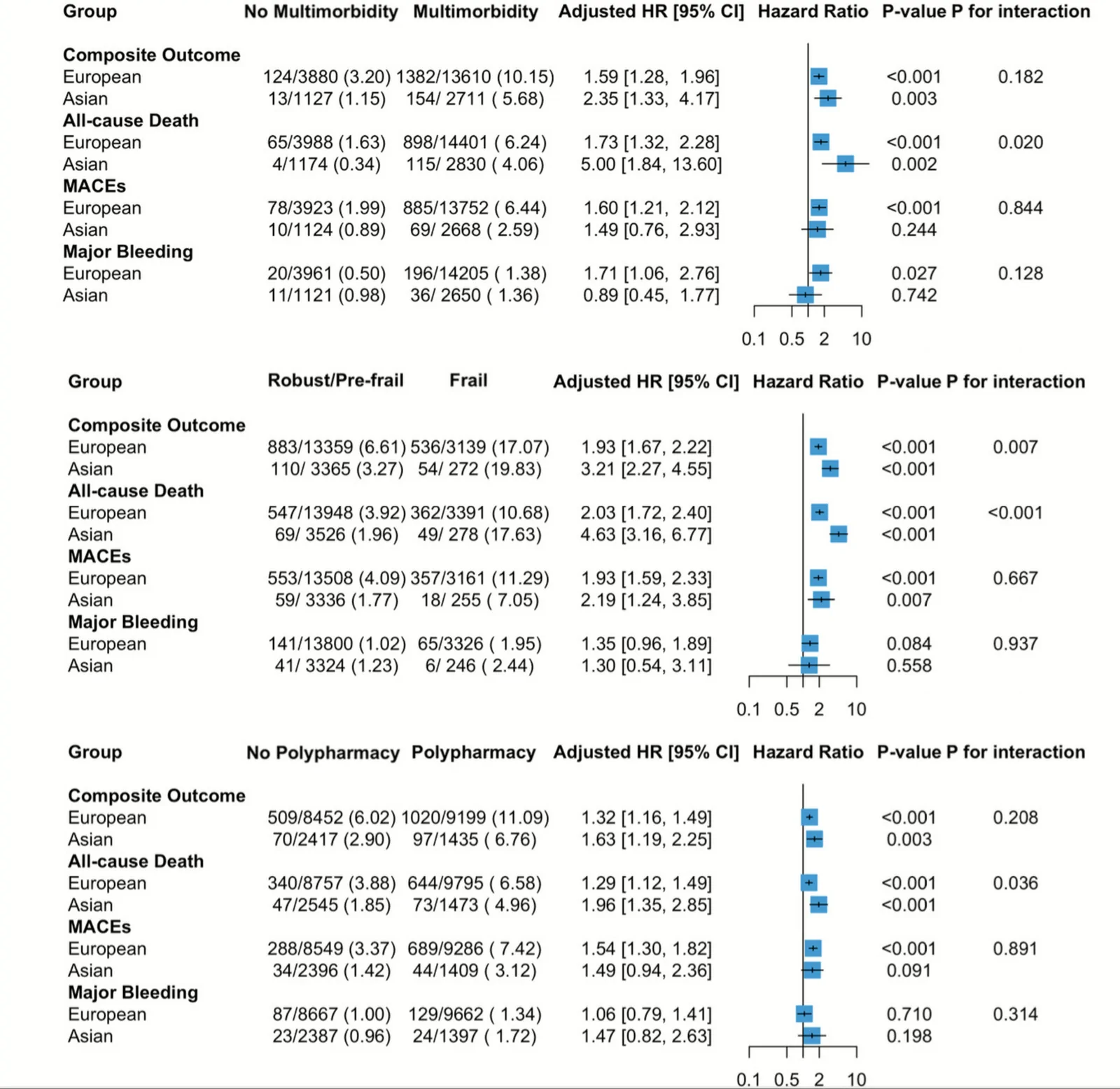
Association between Multimorbidity, Frailty, and Polypharmacy Domains and Adverse Outcomes in European and Asian Patients

Regarding differences between European and Asian patients, Fig. 3 and Supplementary Table S3 show the incidence rates per 100 person-years, aHR, and the results of the interaction analysis between the two groups. For the primary composite outcome, there was a significant interaction effect between frailty and region of enrolment, with frailty having a greater impact among Asian patients compared to the European ones (p_int_ = 0.007). No significant interaction effects were observed for multimorbidity and polypharmacy on the risk of the primary composite outcome.

When evaluating the secondary endpoint of all-cause death, a significant interaction effect with region of enrollment was consistently observed across all domains of clinical complexity. Specifically, a greater risk of all-cause death was found for Asian patients with any clinical complexity domain compared to European individuals. No significant interaction was observed between regions and the risk of MACEs and major bleeding.

### Major adverse outcomes according to burden of clinical complexity

When stratifying patients by the number of clinical complexity domains they fulfilled (i.e., 0, 1, 2, or 3, of multimorbidity, frailty, and polypharmacy), there was a progressive and significant increase in the crude rates of the primary composite outcome, as shown in Supplementary Figure S1. This stepwise increase was consistent across all secondary outcomes [Supplementary Figure S1].

**Fig. 4:**
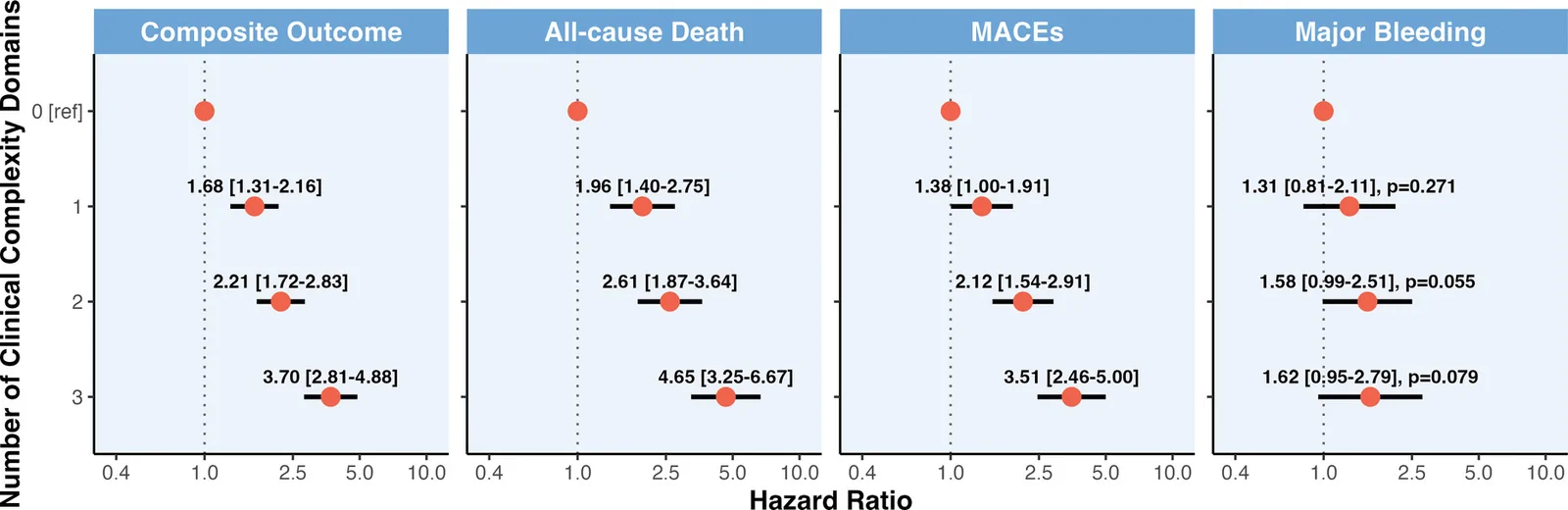
Associations between Number of Clinical Complexity Domains (Multimorbidity, Frailty, and Polypharmacy) and study outcomes

These findings were confirmed in multivariable Cox regression models adjusted for confounders [Fig. 4], where a progressive increase in the hazard of composite outcome events was observed with an increasing burden of clinical complexity domains. Patients with all three multimorbidity, frailty, and polypharmacy domains had a 3.7-fold increased risk of experiencing the primary outcome compared to those without any domain. Similar trends were found for all-cause death and MACEs, while a non-significant trend was noted for major bleeding [Fig. 4].

A significant interaction effect was found between the number of complexity domains and the region of enrolment. Specifically, the impact on the risk of composite outcome events of fulfilling all three clinical complexity domains was greater among Asian individuals compared to their European counterparts (aHR 7.31, 95% CI 3.61–14.83 vs aHR 3.31, 95% CI 2.48–4.43, p_int_ = 0.020; Supplementary Figure S2).

## Discussion

In this analysis of two large, contemporary registries of patients with AF, our main findings are summarized as follows: i) European patients exhibited a higher burden of clinically complex phenotypes compared to Asian patients, with a greater prevalence of multimorbidity, frailty, and polypharmacy domains; ii) the presence of these domains significantly influenced both OAC prescription patterns and adverse clinical outcomes; iii) frailty, in particular, had a more pronounced impact among Asian patients, who were not likely to be prescribed OAC and had a higher risk of adverse events compared to their European counterparts. Also, Asian patients consistently had a significantly higher risk of all-cause death across all domains of clinical complexity.

In recent years, clinical research on AF has increasingly focused on the concept of clinical complexity [27–29], particularly in relation to multimorbidity, frailty, and polypharmacy. These three domains, which frequently coexist in patients with AF, contribute significantly to therapeutic challenges and drug-related adverse outcomes [30–32]. While previous investigations have primarily explored these domains in Western cohorts, data from non-Western populations, especially those from Asia, are limited.

The first key observation from our analysis is the lower prevalence of clinical complexity domains among Asian patients compared to their European counterparts. This represents the first evidence reporting such Asian vs European differences in the prevalence of multimorbidity, frailty, and polypharmacy, which were lower in the Asian cohort. In the general population, both multimorbidity and frailty are more common in Asian and other ethnic minorities than in European older adults [33, 34]. We may hypothesize that differences in our AF cohort compared to the general population can be explained by the higher prevalence of the various comorbidities reported in European AF patients compared to Asian ones. These observations are in line with previous reports from large international registries such as GLORIA-AF and GARFIELD-AF, which showed that Asian AF patients have fewer chronic comorbidities than non-Asian AF patients [35, 36].

Despite being less common, the presence of frailty or polypharmacy in Asian patients was associated with more pronounced consequences in terms of both underuse of OAC therapy and adverse clinical outcomes. Specifically, frailty was associated with significantly lower odds of OAC prescription in Asia compared to Europe, and polypharmacy, while generally associated with increased OAC use, was linked to a smaller effect size in Asian individuals. This pattern of under-prescription with OAC therapy was reported in another cohort in which, compared to non-Asian patients, Asian individuals were less likely to be treated with OAC (OR: 0.23, 95% CI: 0.22–0.25) [35].

Our analysis underlines that this under-treatment may be disproportionately concentrated among clinically complex subgroups, such as frail patients or those with high medication burdens. Several factors may contribute to this phenomenon, ranging from structural barriers, such as delayed non-vitamin K antagonists approval with limited reimbursement, or high out-of-pocket costs [37–39], to physician-related concerns. In particular, the known higher incidence of intracranial hemorrhage among Asians [40] may imply a more cautious approach to anticoagulation, including off-label low-dose OAC use or treatment avoidance, particularly in frail patients. Indeed, a previous systematic review [41] found that fear of bleeding complications may account for 3–25% of OAC discontinuations, while frailty has been reported to contribute to 11–38% of discontinuations. Patient preferences may also play a role: studies in Asian populations have reported that dissatisfaction with treatment, concerns about side effects, and perceived treatment burden were common reasons for discontinuation or refusal of anticoagulation [42, 43].

This pattern of under-prescription may at least partly explain the finding that frail Asian patients in our study experienced a significantly higher risk of the composite outcome of all-cause death and MACE compared to frail European patients, with nearly a threefold increase in adjusted hazard. Similarly, patients presenting with all three domains of multimorbidity, frailty, and polypharmacy faced a markedly elevated risk of adverse events in both regions, but this excess risk was again significantly higher among Asians. These subgroups represent those most susceptible to adverse outcomes, and existing data support the prognostic value of OAC use, which was associated with a lower risk of events across the spectrum of frailty, except in patients with very/extremely high levels of frailty [23]. Therefore, the lower rates of anticoagulation among clinically complex Asian patients, particularly the frailest, may leave them more exposed to preventable complications. This evidence follows similar data reported in other cardiovascular diseases, such as heart failure [44].

In addition to biological or clinical explanations, these differences in outcomes may also reflect disparities in healthcare access and socioeconomic context. In our cohort, Asian patients were more likely to have a lower educational level but less likely to live alone, suggesting distinct social and family structures that may influence healthcare-seeking behavior, disease perception, and adherence to long-term therapies. Moreover, in Asia, and particularly within rural contexts, limited healthcare resources, lower income levels, and reduced availability of specialized care may further contribute to the differences observed with European cohorts [45, 46]. Further prospective registries and region-specific studies will be crucial to better understand these potential relationships and to identify modifiable barriers to equitable care delivery.

The findings of our study have important clinical implications. Multimorbidity, frailty, and polypharmacy in Asian patients appear to define a particularly high-risk group in whom therapeutic inertia or reluctance may carry substantial prognostic costs. Identifying and proactively managing these individuals, through appropriate anticoagulation, close follow-up, and integrated care pathways, such as the ‘Atrial fibrillation Better Care’ (ABC) pathway, may be key to improving outcomes in these vulnerable populations [47]. Current guidelines—including those from Asian societies—recommend an integrated and holistic approach to the management of patients with AF [9, 48]. Data from the APHRS-AF registry indicate that, despite these recommendations, only about 38.9% of AF patients in the region are managed according to such integrated care pathways [49].

Supporting this, the mAFA (mobile Atrial Fibrillation Application) cluster randomized trial conducted across 40 centers in China demonstrated a 61% reduction in the composite outcome of ischemic stroke, systemic thromboembolism, death, and rehospitalization with the mAFA intervention compared to usual care (1.9% vs. 6.0%; HR: 0.39, 95% CI 0.22–0.67; P < 0.001) [50]. The more recent MIRACLE-AF trial showed that among Chinese AF patients living in rural villages, delivery of the ABC pathway by village doctors using a telemedicine-based approach was effective in increasing implementation of the ABC pathway, leading to a significant reduction in major adverse outcomes [51].

One post-hoc analysis of the mAFA trial showed that the effect of the intervention on the primary outcome appeared greater in the low morbidity phenotype (HR, 0.08, 95% CI 0.02–0.33) compared to the more complex phenotype (HR, 0.68, 95% CI 0.37–1.24), with a statistically significant interaction (p_int_ = 0.004) [52]. These findings suggest that the ABC pathway improved prognosis across different comorbidity phenotypes, even if patients with more complex phenotypes need further efforts to improve their outcomes, given their high baseline risk of adverse events.

### Study limitations

Our study has several limitations that should be acknowledged. First, this was a retrospective analysis of prospectively collected data from two observational registries, and inherent biases or residual confounding cannot be excluded. Although we adjusted for a wide range of clinical variables, unmeasured or unknown factors may still have influenced our findings. Second, the definition and operationalization of clinically complex domains—including multimorbidity, frailty, and polypharmacy—relied on available registry data, and some degree of misclassification or underreporting may have occurred, particularly for frailty, which was assessed using a retrospective frailty index. Third, differences in data collection protocols and follow-up durations between the European and Asian registries may have introduced heterogeneity, which could affect the comparability of outcomes.

In addition, a potential survivor bias cannot be ruled out, as patients included in the registries were those surviving long enough to be enrolled and evaluated at baseline.

Regarding missing data, no specific imputation techniques were applied; however, the overall rate of missing variables was very low (< 1% for all variables included in the logistic and Cox models), reflecting the high completeness of the databases. Therefore, it is unlikely that missing data significantly influenced the study results.

Finally, our cohorts of patients are representative of individuals recruited from European and Asian countries; as such, the observed differences may not necessarily reflect ethnic or biological variability but could be influenced by the characteristics of the two registries themselves. In addition, differences in follow-up durations between the registries, as defined in their original protocols, represent a further limitation and warrant caution when interpreting outcome comparisons.

## Conclusion

Multimorbidity, frailty, and polypharmacy domains display distinct epidemiological patterns between European and Asian patients with atrial fibrillation. Frailty, in particular, was associated with a lower likelihood of oral anticoagulant prescription, especially among Asian patients. Importantly, the presence of multimorbidity, frailty, or polypharmacy was linked to a higher risk of adverse outcomes, with this excess risk being more pronounced in the Asian population.

## Supplementary Information

Below is the link to the electronic supplementary material.

ESM 1 (DOCX 390 KB)

## Data Availability

The data underlying this article will be shared on reasonable request to the corresponding author.
