# Supplementary material for: Multimorbidity, frailty and polypharmacy in European and Asian patients with atrial fibrillation: a comparison of two regional prospective observational registries
Source: GeroScience. 2025 Dec 4;48(5):6871–85. doi: 10.1007/s11357-025-02026-5 (PMC13601450; doi:10.1007/s11357-025-02026-5)
Supplement: Supplementary file 1 [file 11357_2025_2026_MOESM1_ESM.docx]

**Multimorbidity, frailty and polypharmacy in European and Asian Patients with Atrial Fibrillation: A Comparison of Two Regional Prospective**

**Observational Registries**

**Brief Title:** Multimorbidity, frailty and polypharmacy in AF Patients

**Supplementary TABLES**

**Table S1: Items Included into the Frailty Index**

| ***1. Vital Signs Domain*** | **DEFINITION** | **DEFICIT**  **VALUE** |
| --- | --- | --- |
| Systolic Blood Pressure | ≥140 mmHg | 1 |
| Diastolic Blood Pressure | ≥90 mmHg | 1 |
| Heart Rate | ≥110 bpm | 1 |
| Body Mass Index | <18.5 kg/m^2^  25.0-29.9 kg/m^2^  ≥30.0 kg/m^2^ | 1  0.5  1 |
| ***2. Comorbidities Domain*** |  |  |
| Hypertension | Present | 1 |
| Diabetes Mellitus | Present | 1 |
| Lipid Disorder | Present | 1 |
| Coronary Artery Disease | Present | 1 |
| Heart Failure | Present | 1 |
| Valvular Disease | Present | 1 |
| Cardiomyopathy | Present | 1 |
| Pulmonary Arterial Hypertension | Present | 1 |
| Peripheral Artery Disease | Present | 1 |
| Previous Thromboembolic Events | Present | 1 |
| Previous Hemorrhagic Events | Present | 1 |
| Hyperthyroidism | Present | 1 |
| Hypothyroidism | Present | 1 |
| Chronic Kidney Disease | Present | 1 |
| Liver Disease | Present | 1 |
| Chronic Obstructive Pulmonary Disease | Present | 1 |
| Obstructive Sleep Apnoea Syndrome | Present | 1 |
| Dementia | Present | 1 |
| Anaemia | Present | 1 |
| Malignancy | Present | 1 |
| ***3. Symptoms Domain*** |  |  |
| Palpitations | Present | 1 |
| Syncope | Present | 1 |
| Shortness of Breath | Present | 1 |
| Chest Pain | Present | 1 |
| General not-Well Being | Present | 1 |
| Dizziness | Present | 1 |
| Fatigue | Present | 1 |
| Fear | Present | 1 |
| Other Symptoms | Present | 1 |
| ***4. Function/Autonomy Domain***  *(EQ-5D-5L Questionnaire)* |  |  |
| Mobility | No Problems  Slight Problems  Moderate Problems  Severe Problems  Unable to Walk | 0  0.25  0.5  0.75  1 |
| Self-Care | No Problems  Slight Problems  Moderate Problems  Severe Problems  Unable to Wash/Dress | 0  0.25  0.5  0.75  1 |
| Usual Activities | No Problems  Slight Problems  Moderate Problems  Severe Problems  Unable to Usual Activities | 0  0.25  0.5  0.75  1 |
| Pain/Discomfort | No Pain/Discomfort  Slight Pain/Discomfort  Moderate Pain/Discomfort  Severe Pain/Discomfort  Extreme Pain/Discomfort | 0  0.25  0.5  0.75  1 |
| Anxiety/Depression | None  Slight Anxious/Depressed  Moderate Anxious/Depressed  Severely Anxious/Depressed  Extremely Anxious/Depressed | 0  0.25  0.5  0.75  1 |
| ***5. Biomarkers Domain*** |  |  |
| Creatinine Clearance (CKD-EPI) | <60 mL/min | 1 |
| Haemoglobin | <13 mg/dL for males  <12 mg/dL for females | 1 |

**Table S1**. Pharmacological treatments among Clinical Complexity Domains and registries

|  | **Multimorbidity** | | **Frailty** | | **Polypharmacy** | |
| --- | --- | --- | --- | --- | --- | --- |
| n | **EORP-AF**  **N=8655** | **APHRS**  **N=3230** | **EORP-AF**  **N=2172** | **APHRS**  **N=340** | **EORP-AF**  **N=5927** | **APHRS**  **N= 1696** |
| Antiplatelet therapy, n (%) | 1942 (22.5) | 550 (17.0) | 667 (30.8) *_0.660_* | 100 (29.4) *_0.660_* | 1509 (25.5) **_0.017_** | 383 (22.6) **_0.017_** |
| VKAs, n (%) | 4662 (53.9) | 784 (24.3) | 1234 (56.9) | 102 (30.0) | 3529 (59.5) | 487 (28.7) |
| NOACs, n (%) | 2878 (33.3) | 1949 (60.3) | 569 (26.2) | 157 (46.2) | 1922 (32.4) | 1029 (60.7) |
| ACE-inhibitors, n (%) | 4088 (47.4) | 544 (16.9) | 1077 (49.9) | 70 (20.6) | 3324 (56.1) | 452 (26.7) |
| ARBs, n (%) | 1838 (21.3) | 971 (30.1) | 472 (21.8) | 114 (33.5) | 1497 (25.3) | 734 (43.3) |
| Beta-blockers, n (%) | 6192 (71.8) | 1795 (55.8) | 1616 (74.7) | 205 (60.5) | 4759 (80.3) | 1208 (71.2) |
| Aldosterone blocker, n (%) | 1888 (21.9) | 277 (8.6) | 684 (31.7) | 51 (15.0) | 1808 (30.5) | 248 (14.6) |
| Diuretics, n (%) | 5133 (59.5) | 893 (27.7) | 1563 (72.4) | 177 (52.4) | 4494 (75.8) | 741 (43.7) |
| Digoxin, n (%) | 1418 (16.4) **_0.003_** | 456 (14.2) **_0.003_** | 455 (21.1) *_0.169_* | 60 (17.6) *_0.169_* | 1311 (22.1) *_0.518_* | 362 (21.3) *_0.518_* |
| Calcium channel blockers, n (%) | 1589 (18.4) | 867 (26.9) | 430 (19.9) | 106 (31.2) | 1426 (24.1) | 617 (36.4) |
| Non-DHP CCB, n (%) | 472 (5.5) | 455 (14.1) | 127 (5.9) | 62 (18.2) | 361 (6.1) | 331 (19.5) |
| Statin, n (%) | 4201 (48.7) *_0.400_* | 1538 (47.8) *_0.400_* | 1163 (53.8) | 227 (67.2) | 3537 (59.7) | 1162 (68.5) |
| Oral antidiabetics, n (%) | 1609 (18.6) | 699 (21.8) | 586 (27.1) **_0.005_** | 116 (34.6) **_0.005_** | 1464 (24.7) | 526 (31.0) |
| Insulin, n (%) | 593 (6.9) | 116 (3.6) | 292 (13.5) *_0.618_* | 42 (12.4) *_0.618_* | 568 (9.6) | 105 (6.2) |

Legend: *all comparisons depict statistically significant differences at p, except those that were explicitly reported in the subscripts, in which Italic characters depict non-significant differences and Bold characters depict significant differences.

Multimorbidity is defined as >= 2 comorbidities

Polypharmacy is defined by assuming 5 or more drugs

Frailty is defined by frailty index values > 0.20.

ACE, angiotensin-converting enzyme; ARB, angiotensin receptor blocker; CCB, calcium channel blocker; DHP, dihydropyridine; NOAC, Non-Vitamin K oral anticoagulant; VKA, vitamin K antagonist.

**Supplementary Table S3.** Incident rate per 100 person-years with 95% CI of adverse events for Multimorbidity, Frailty and Polypharmacy, stratified by region of enrolment

|  | **No Multimorbidity** | **Multimorbidity** | **Robust/Pre-Frail** | **Frail** | **No polypharmacy** | **Polypharmacy** |
| --- | --- | --- | --- | --- | --- | --- |
| **Composite Outcome** |  |  |  |  |  |  |
| European | 3.20 [2.66 -3.81] | 10.15 [9.63 -10.70] | 6.61 [6.18 -7.06] | 17.07 [15.66 -18.58] | 6.02 [5.51 -6.57] | 11.09 [10.42 -11.79] |
| Asian | 1.15 [0.61 -1.97] | 5.68 [4.82 -6.65] | 3.27 [2.69 -3.94] | 19.83 [14.89 -25.87] | 2.90 [2.26 -3.66] | 6.76 [5.48 -8.25] |
| **All-cause Death** |  |  |  |  |  |  |
| European | 1.63 [1.26 -2.08] | 6.24 [5.83 -6.66] | 3.92 [3.60 -4.26] | 10.68 [9.60 -11.83] | 3.88 [3.48 -4.32] | 6.58 [6.08 -7.10] |
| Asian | 0.34 [0.09 -0.87] | 4.06 [3.35 -4.88] | 1.96 [1.52 -2.48] | 17.63 [13.05 -23.31] | 1.85 [1.36-2.46] | 4.96 [3.89 -6.23] |
| **Mace** |  |  |  |  |  |  |
| European | 1.99 [1.57 -2.48] | 6.44 [6.02 -6.87] | 4.09 [3.76-4.45] | 11.29 [10.15 -12.53] | 3.37 [2.99 -3.78] | 7.42 [6.88 -7.99] |
| Asian | 0.89 [0.43 -1.64] | 2.59 [2.01 -3.27] | 1.77 [1.35 -2.28] | 7.05 [4.18 -11.15] | 1.42 [0.98 -1.98] | 3.12 [2.27 -4.19] |
| **Major Bleeding** |  |  |  |  |  |  |
| European | 0.50  [0.31 -0.78] | 1.38 [1.19 -1.59] | 1.02 [0.86 -1.20] | 1.95 [1.51 -2.49] | 1.00 [0.80 -1.24] | 1.34 [1.11 -1.59] |
| Asian | 0.98  [0.49 -1.76] | 1.36 [0.95 -1.88] | 1.23 [0.89 -1.67] | 2.44 [0.89 -5.30] | 0.96 [0.61 -1.45] | 1.72 [1.10 -2.56] |

**Supplementary FIGURES**

**Supplementary Figure S1.** Distribution of outcomes according to the number of clinical complexity domains

**Supplementary Figure S2.** Cox regression showing the interaction between the number of clinical complexity domains and the registry of enrollment
